# Conventional approaches to indicators and metrics undermine urban climate adaptation

**DOI:** 10.1038/s42949-025-00310-z

**Published:** 2025-12-14

**Authors:** Marta Olazabal, Andressa V. Mansur, Samraj Sahay, Laura Helmke-Long, Massimiliano Granceri Bradaschia, Ane Villaverde, Leire Garmendia, Prince Dacosta Aboagye, Ayyoob Sharifi, Obed Asamoah, Patricia Mwangi, William Lewis, Borja Izaola, Ellie Murtagh, Ira Feldman

**Affiliations:** 1 Basque Centre for Climate Change (BC3), Leioa, Spain; 2 IKERBASQUE, Basque Foundation for Science, Bilbao, Spain; 3 Independent Researcher, New Delhi, India; 4 Indiana University, O’Neill School of Public and Environmental Affairs, Bloomington, IN USA; 5 University Iuav of Venice, Department of Architecture and Arts, Venice, Italy; 6 University of the Basque Country (UPV/EHU), School of Engineering, Bilbao, Spain; 7 Nagoya University, Graduate School of Civil and Environmental Engineering, Nagoya, Japan; 8 Hiroshima University, The IDEC Institute & Network for Education and Research on Peace and Sustainability (NERPS), Hiroshima, Japan; 9 School of Architecture and Design, Lebanese American University, Beirut, Lebanon; 10 Ipsos Centre for Development Research and Evaluation, Ipsos, Accra, Ghana; 11 Kenyatta University, Department of Spatial and Environmental Planning, Nairobi, Kenya; 12 University of the Basque Country (UPV/EHU), Bilbao, Spain; 13 British Red Cross, West Point House, Edinburgh, United Kingdom; 14 Adaptation Leader, Washington, DC USA

**Keywords:** Climate sciences, Ecology, Ecology, Environmental sciences, Environmental social sciences

## Abstract

Measurement is essential for effective adaptation management and operation, and indicators and metrics (I&M) have a pivotal role. Surprisingly, systematic efforts to assess advances in the provision of adaptation I&M are scarce, and those that do exist often lack in-depth analysis of the types, characteristics, and applicability of the collected information. Here, we analyse 137 publications and 901 I&M sourced in the scientific literature (2007–2022) to measure adaptation to climate change in urban areas where governments are increasingly placing efforts to prepare populations and infrastructures. A lack of common terminology, standardisation, and guidelines has resulted in a field that is complex to track and understand. This complexity has led to a fragmented methodological landscape, marked by diverse, context-dependent, and occasionally conflicting approaches to the development of I&M. We argue that conventional approaches to I&M are largely inadequate and must better emphasise quantifiability, long-term assessment, and alignment with policy objectives.

## Introduction

While tracking emission pledges currently dominates international conversations^1^, the evaluation of progress on climate change adaptation has also become a hot topic across scientific and policy arenas, and at multiple levels of governance^2^. Measuring adaptation progress is essential for understanding adaptation needs, accounting for actions, and assessing their effectiveness and efficiency^3^. Measurement is also important to evaluate positive and negative impacts and the equity of adaptation actions^4^. Measurement helps learning and improves future adaptation processes, allowing for comparisons and benchmarking. Finally, it helps attract political momentum and funding, as well as to understand the relationship of adaptation with other societal, climate or biodiversity challenges^5–7^. The conceptual and empirical adaptation literature is vast, scattered and difficult to track for many reasons. First, there is an ambiguous use of language - e.g. interchangeably using “climate resilience,” “climate adaptation,” “climate vulnerability”, or risk reduction” to refer to states of better preparedness and unclear connections with adaptation monitoring, evaluation, reporting, and learning (MERL) objectives and stages. Second, climate change adaptation-related literature spans across multiple disciplines and sectors^8,9^. Third, and connected to the above, while attempts have been made^10–14^, shared frameworks for adaptation measurement research and practice are lacking. Much attention is being directed to identifying ways to measure progress towards the Global Goal on Adaptation^15–18^, however, up to now, there is no good understanding of the advances in the field of adaptation measurement and, in particular, the means of measurement—indicators and metrics (I&M), across the adaptation cycle, scales and sectors.

As a result of the context-specific nature of adaptation needs and the absence of universal effects from adaptation actions, the field of adaptation measurement has moved forward under simplified assumptions. For urban adaptation, for instance, the accountability and quality assessment of adaptation plans and policies have typically been used as proxies for progress^19–23^, overlooking their symbolic dimension^24^ and lack of financing or implementation^25^. The scant attention to I&M has largely been theoretical or too context- or sector-specific^26–32^. Few studies have comprehensively and systematically analysed the state of the art of urban climate change adaptation I&M. Several studies have made initial steps toward this goal. Arnott et al.^11^ provided an analysis of 43 urban adaptation I&M documents gathered from grey literature developed by governments, boundary organisations and sponsors. Salehi et al.^30^ performed a systematic review and extracted 176 adaptation I&M from 59 mainly academic sources. Goonesekera and Olazabal^12^ coded 1971 I&M sourced in 11 local adaptation plans. And, finally, as an intervention-specific example, Goodwin et al.^7^ conducted a review of 750 I&M indicators drawn from reports on nature-based adaptation solutions in cities around the world.

To advance this effort, we systematically review adaptation I&M in the scientific literature—a vast and scattered body of work that necessitates tailored documentation protocols. Beyond cataloguing types of I&M, we conduct an in-depth analysis of the theoretical frameworks, characteristics, and applicability of I&M, thereby extending current documenting approaches. We focus on I&M specifically targeted to measure climate change adaptation in urban areas, where governments around the globe are increasingly making efforts to prepare populations and infrastructures for the impacts of climate change through plans and policies. This is a pioneering effort that has been designed to understand four key aspects: (i) the nature and geography of existing empirical research work, (ii) the typology of climate impacts and adaptations covered, (iii) the landscape of I&M currently proposed, and (iv) their intended users and uses. We systematically identify 838 publications from the openly accessible and multi-sourced LENS database. Across a set of 137 publications capturing indicators that measure climate change adaptation in urban areas (see Tables S1 and S2), we collect and examine 901 I&M (including indices). The publications reviewed are dated from 2007 to 2022, with 70% published after 2016, reflecting the increased attention to I&M after the Paris Agreement in 2015. The evidence gathered aligns with the cut-off date of the IPCC’s Sixth Assessment Report, offering a timely assessment foundation for the next cycle.

## Results

### Geography of the studies

**Fig. 1:**
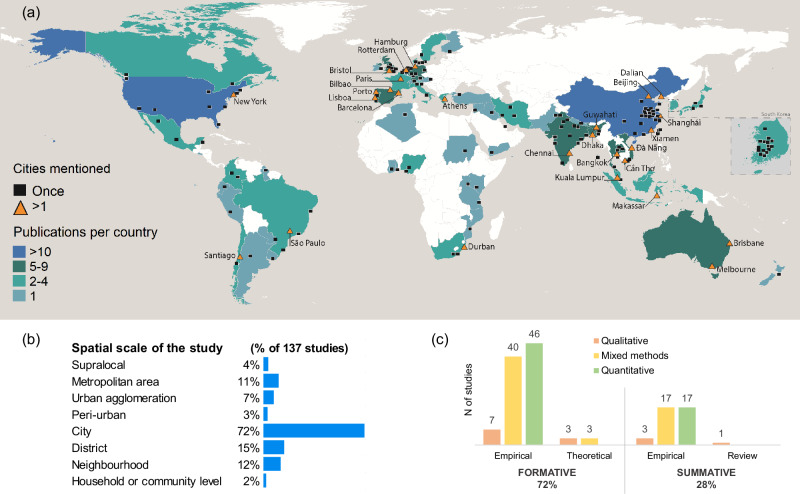
Analysis of 137 publications. **a** Map showing the cities and countries that have been used as application sites for adaptation indicators and metrics. The map shows the cities mentioned in the different publications and the number of publications connected to countries (as validation sites). **b** Spatial scale of the studies as a percentage of the total number of publications reviewed. **c** Type of assessments and types of methods (formative and summative, theoretical, empirical and review, and quantitative, qualitative and mixed methods). The spatial scale categories used in this analysis (see **b**) were derived from the descriptions found in the reviewed studies and are presented in this figure in order of increasing spatial extent. As an illustration, the term urban agglomeration denotes several municipalities considered jointly, while the metropolitan scale is used when such an area has an official administrative recognition as a metropolitan area.

The vast majority of studies are empirical (95% of 137). Few studies are conceptual/theoretical (4%) or review works (1%). The empirical body of work focuses on specific geographic regions and cities and discusses the applicability of proposed indicators. Most I&M are applied in Asian (42%) and European (31%) cities, followed by North American (16%), Latin American (12%), African (11%) and Oceanian (5%) (see Fig. 1a). In a few cases (9%), cities from different world regions are looked at in combination, but the application of I&M is addressed generally with regional exclusivity. Studies encompass a diversity of spatial scales, from the supralocal to the household level. Many I&M are not confined to a single scale, but are relevant across multiple spatial levels. These multi-scale I&M were coded at all relevant levels. A substantial majority (72%) mention addressing the city as a whole. Twenty-five per cent of studies mention addressing scales beyond the city level while still assessing urban adaptation interventions (peri-urban, urban agglomerations, metropolitan and supralocal), and 29% of studies focused on scales often below the city level, including district, neighbourhood, and household or community level (Fig. 1b).

### Type of assessments and methods

Generally, I&M are used in formative assessments (i.e., to understand baseline conditions and vulnerabilities) or in summative assessments (i.e., to understand effectiveness, efficiency, and performance)^33^. A significant share of our sample (72%) (Fig. 1c) adopts a formative approach, where I&M are used to identify specific sectors, populations, or spatial areas where adaptation capacities need to be built or increased. The literature, however, often remains ambiguous about whether and how assessments of adaptation needs (typically conducted by mapping changes in vulnerabilities, risks, or adaptive capacities) are linked to MERL processes. For example, although indicators for tracking evolving vulnerabilities are commonly proposed, there is a lack of corresponding guidance on how these results will be evaluated, interpreted, and used to refine, adjust, or act upon existing adaptation interventions. Alternatively, summative studies (28%) look into the assessment of implemented adaptations and propose I&M to monitor, evaluate, report, and learn from specific urban adaptation processes and actions on the ground.

To build I&M, quantitative (46%) and mixed methods (44%), such as survey data analysis, statistical analysis, and data modelling, are predominant across empirical studies, both for formative and summative assessments (Fig. 1c). The use of qualitative approaches, like in-depth interviews, focus group discussions, case studies, thematic analysis, observations, and content analysis to build I&M is less common (10%).

### Disciplines and theoretical frameworks behind I&M

**Fig. 2:**
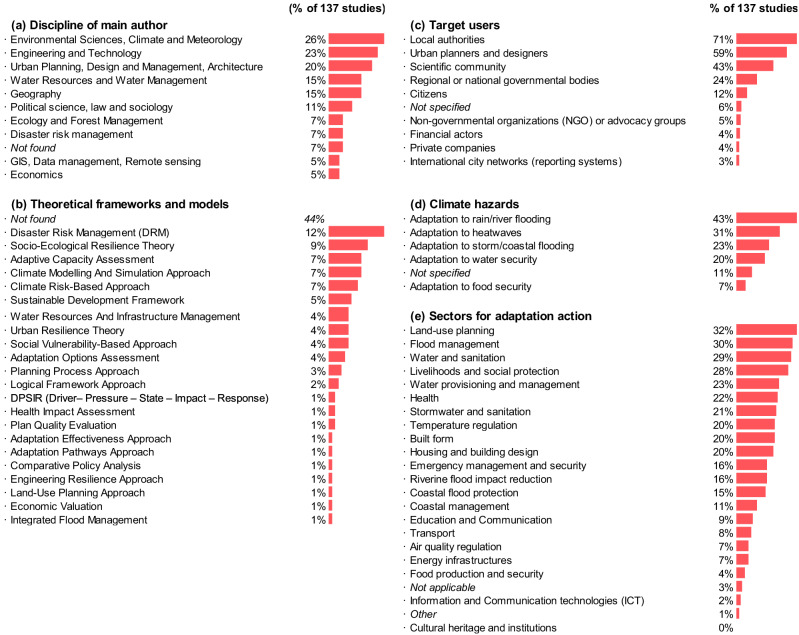
Analysis of 137 publications. **a** Discipline of main author (frequency) collected from institutional profiles and social media accounts; **b** Theoretical frameworks and models (frequency) inferred from publication records; **c** Target users inferred from publication records; **d** Climate hazards (frequency) explicitly mentioned in the publication records, and **e** Types of adaptation measures (frequencies) inferred in publication records. Categories for climate hazards and types of adaptation measures in urban areas correspond to those used in the 6th Assessment Report (AR6) of the Intergovernmental Panel on Climate Change (IPCC)^19,22^.

Technical areas such as environmental sciences, climate and meteorology (26%), engineering and technology (23%) and urban planning, design, management and architecture (20%) (Fig. 2a) address urban adaptation I&M more frequently. By contrast, we found less prevalence of social science and interdisciplinary areas such as geography (15%), economics (5%), political science, law and sociology (11%) and disaster risk management (7%).

Around 44% of the studies do not mention any theoretical background or model used to guide or frame the proposal of urban adaptation I&M. The studies that identify a theoretical framework show a massive degree of dispersion (Fig. 2b). The most common approach used across studies is disaster risk management (12%), adaptive capacity assessments (7%), climate modelling and simulation approaches (7%), socio-ecological resilience theory (9%) and climate risk-based approaches (7%) follow closely. None of the studies referred to specific MERL frameworks^10,11,13,27^.

### Target users

The primary audience or target users of the study are rarely explicitly mentioned or justified. Despite this ambiguity, the data indicate that local authorities (71% of the studies), urban planners and designers (59%) and the scientific community (43%) were most often implicitly identified as the intended recipients of the studies (Fig. 2c). Regional or national governmental bodies follow closely (24%). Other local actors, such as citizens (12%), non-governmental organisations (NGO) or advocacy groups (5%), financial actors (4%), private companies (4%), or international city networks (3%), are only occasionally considered as users for urban adaptation I&M.

### Types of hazards and adaptations

In line with global urban adaptation responses^3^, our review reveals that the most frequently considered hazard is rain/river flooding (43%), followed by storm/coast flooding (32%) and heatwaves (23%), with less attention paid to water security (20%) and food security (7%) (Fig. 2d). The I&M studies in our sample look at a wide range of adaptation measures. The most popular are land-use planning (32%), flood management (30%), water and sanitation (29%), and livelihoods and social protection (28%) (Fig. 2e). The least focused on are education and communication (9%), air quality regulation (7%), food production and security (4%), information, communication and technology (ICT) (2%), energy infrastructures (7%) and transport (8%). Among the IPCC categories^19^, cultural heritage and institutions gather zero attention.

### Types of indicators and metrics (I&M)

**Fig. 3:**
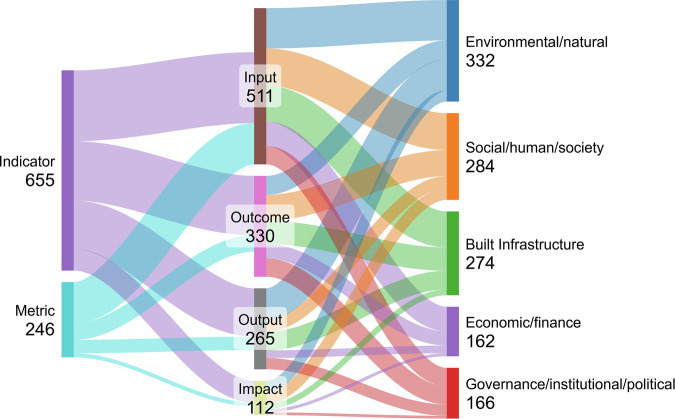
Sankey diagram showing the analysis of 901 urban adaptation Indicators and Metrics (I&M). The diagram visualises connections between the level of detail, type, and dimension of I&M (from left to right). The level of detail indicates the number of indicators and metrics at each level, relative to the total of 901 I&M. The type of I&M includes input, output, outcome, and impact, with values ordered according to their respective frequencies. Dimension refers to thematic areas (e.g., environmental, social, economic), ordered by frequency. As I&M can be multidimensional, totals may exceed 901.

We gathered 901 I&M from 137 studies and distinguished between single I&M and composite I&M (typically, indices composed of more than one indicator or metric). Only 15% of the I&M are classified as indices (e.g. “Adaptive Capacity Index,” “Integrated Urban Resilience Index” or “Heat Vulnerability Index”). The remaining are classified as single I&M, for example, “Per cent Green Open Space,” “Diversity of Renewable Energy,” and “Increased Flood Insurance Coverage”. We analyse the indices in their composite form. We also distinguish two categories of I&M depending on their tangibility: indicators and metrics^11^. While indicators can be general and unspecific (e.g. population vulnerability), metrics represent more detailed, tangible measurements (e.g. number of trees). Concurring with previous studies^12^, a significant majority of I&M (73%) are identified as “indicators”, encompassing both single and composite forms. The remaining 27% are expressed as “metrics”, encompassing only single forms (Fig. 3).

We categorise I&M into four types: input, output, outcome, and impact^10,12,34^. The distribution reveals important insights into current measurement priorities in urban climate adaptation. A high proportion of input metrics (41%) indicates a strong focus on tracking resources and efforts invested in adaptation processes. In contrast, outputs (22%) and outcomes (28%) receive comparatively less attention, despite their role in capturing tangible results and immediate effects of adaptation strategies. Most notably, impact I&M account for only 9%, highlighting a significant gap in the measurement of long-term, systemic consequences of adaptation interventions. This imbalance becomes even more pronounced when considering metrics alone, where inputs rise to 57%, further underscoring a dominant focus on what is being invested, rather than on what is being achieved (Fig. 3).

I&M vary between the dimensions they look at^12,31^. Our study finds the environmental/natural dimension (27%) to be most prevalent (Fig. 3). This dimension encompasses a wide range of critical environmental variables, including, for example, those related to green or blue space, temperature change, sequestration capabilities, flooding, and biodiversity-related variables. The social/human/society dimension (23%) includes aspects such as knowledge, perception, community preparedness, or educational activities. Built infrastructure (22%) looks at resilient urban structures, materials, properties, and other characteristics in urban climate adaptation planning and design. The governance/institutional/policy dimension (14%) and the economic/finance dimension (13%) are less explored. Our data further shows that for single I&M, 80% encompass one dimension and 20% have two dimensions. The composite I&M are more multidimensional in nature (23% two dimensions, 11% three, 11% four, and 10% five). I&M are represented across all dimensions, but progress indicators (outcomes and impacts) are more prevalent in the governance/institutional/political and social/human/society dimensions relative to process indicators (input and outputs) (Fig. 3).

### Applicability and feasibility of urban adaptation I&M

**Fig. 4:**
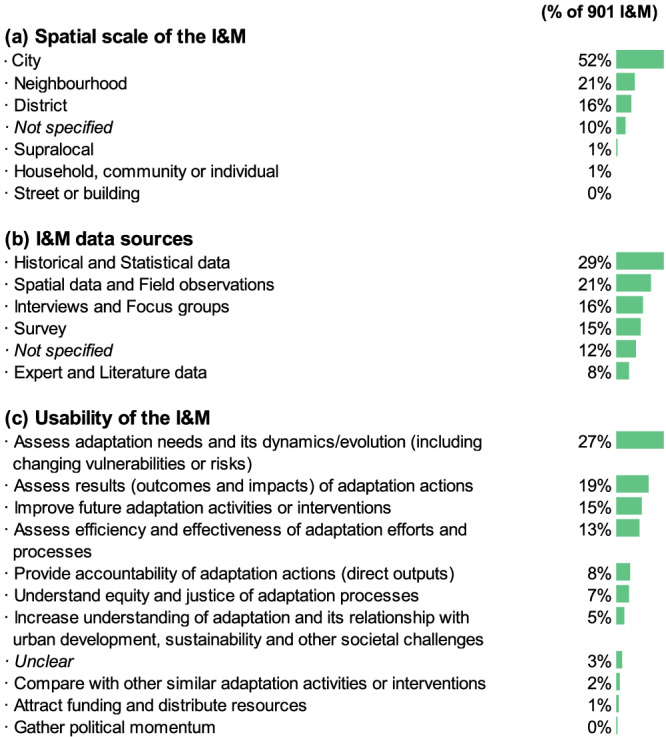
Analysis of 901 I&M. **a** Spatial scale (frequency); **b** Data sources (frequency); **c** Usability of each I&M (frequency) inferred from the publication records (based on Turner et al.^53^).

In 75% of the cases, I&M are not linked to specific adaptation measures regardless of the composite nature or its level of detail. Of the remaining 25%, more than half reference measures related to environmental/natural or built/infrastructure dimensions. Our analyses show that the applicability of the I&M continues to be most prevalent at the city level (52%) (Fig. 4a). The supralocal, household, and community levels receive less attention (1% or less, respectively), with none at the metropolitan or urban agglomeration scales. The remaining efforts focus on the neighbourhood (21%) and district (16%) levels. In 10% of cases, the scale to which the I&M is applicable is not clearly defined.

A large majority of I&M are based on historical and statistical data (29%), followed by spatial data and field observations (21%), interviews and focus groups (16%), surveys (15%) and expert and literature data (8%) (Fig. 4b). The source is not specified in only 12% of cases. Our data also shows that, in around 48% of the cases, the I&M lack a specified unit of measurement. The units of measurement include percentages of some criteria (26%); length, area, or volume (18%); or binary data (i.e., yes or no) (13%). Higher levels of detail imply higher feasibility in application. While 97% of the metrics have an associated unit of measurement, only 35% of the indicators do. Types of I&M have varying levels of associated units of measurement: outputs (62%), outcomes (48%), inputs (53%), and impacts (37%). For I&M looking at governance/institutional/political aspects, only 33% specify units of measurement, in contrast to I&M looking at economic/finance aspects (59%). From the I&M that have associated units of measurement, only 3% specified the required frequency of measurement (80% of which are measured annually).

Finally, we also collect information regarding the purposes behind measuring adaptation for each I&M (Fig. 4c). This information is often ambiguous and requires an interpretation by the analyst. For sets of I&M, this information is normally very similar but not always the same. In most cases (27%), the collected data can potentially be used to assess adaptation needs and their dynamics/evolution. Other purposes include evaluating the results of adaptation actions (outcomes and impacts) (19%), improving future adaptation activities or interventions (15%), and assessing the efficiency and effectiveness of adaptation efforts and processes (13%). Less attention is paid to other important reasons to measure the progress of adaptation, such as comparing with other similar adaptation activities or interventions (2%), attracting funding and distributing resources (1%), gathering political momentum (0.4%), or increasing understanding of adaptation and its relationship with other societal challenges (5%).

## Discussion

In our work, we have adopted a flexible and comprehensive coding approach that, as far as possible, reflects the complexity and cross-scale nature of adaptation and its associated literature. The exercise shows, however, that extracting detailed data on adaptation I&M is a complex task. First, the language used in this scholarly field is often ambiguous and used lightly. For example, the terms indicator and metric are often used in the absence of a concrete form of measurement, i.e. units of measurement, aligning with previous reviews^7,11,12^. In our coding process, we found that the same applies to references to adaptation “evaluation,” “measurement,” and “assessment” that often do not refer to an analytical process to determine the results of an adaptation action. Secondly, we have observed a lack of clarity when it comes to explaining the theoretical framework and application of I&M. The definition of adaptation I&M, both in science and policy, requires a conceptual framework that not only provides guidance and a theory of change but also a shared language^27^. Our results, however, show a lack of conceptual basis for I&M as a result of weak engagement with the theoretical foundations of social vulnerability, resilience, and adaptation research^35^. Arguably, this might be the reason why we find such a complex body of literature that is difficult to track, understand, and apply.

One key consequence of the fragmentation in the adaptation I&M field is a weak potential to standardise MERL frameworks, indicators, and targets for assessing climate adaptation. International initiatives such as ISO 37123 (Indicators for Resilient Cities) and ISO 14090 (Adaptation to Climate Change—Principles, Requirements and Guidelines) illustrate these efforts, but remain generic and only partially applicable to urban adaptation I&M^36,37^. However, despite the widespread advocacy for universal I&M, scholars also warn about the negative consequences of overlooking the political context of standardisation^33,38^. Our review shows that there is an immense diversity of I&M used to measure adaptation efforts at the urban level alone. This diversity reflects the attention to the local nature of adaptation, but it also creates difficulties for comparability, benchmarking, reporting and ultimately, effective adaptation planning, implementation and management, as found elsewhere^39^. Moreover, it may overwhelm the limited resources of local authorities who have to deal with a large number of indicators in their broader sustainability assessment efforts^19^.

This review finds that most empirical work focuses on the city scale and on case studies from European and Asian countries, highlighting a need for more research at sub-city scales and in underrepresented regions. It also shows greater attention to floods and heatwaves, likely due to a strong emphasis on land use, housing, urban planning, and water management sectors across the publications. Despite this, we found a widespread lack of clear links between I&M and specific adaptation measures, limiting their use in MERL practice. The review also shows a sectoral bias, with areas like cultural heritage and institutions largely overlooked (Fig. 2e), suggesting challenges in interdisciplinary collaboration.

Beyond urban planning and geography, our data shows that expertise in social sciences is not leading work in the field. This reflects existing trends in the broader adaptation field, where environmental-related disciplines are dominant^9^. While reasons for this might be diverse and difficult to track^9,40^, this brings into question whether the long-standing experience in MERL in political sciences and business management research areas^41,42^ has had a chance to influence this emergent academic field. It also questions whether and how critical social and economic aspects of vulnerability, equity and justice are being connected to approaches for monitoring and evaluation to avoid, for example, maladaptive practices, and explains the lack of attention to these issues in current evaluation practice^4,43,44^. While quantitative works are more prevalent than qualitative ones, the use of mixed methods is an extended practice, which we interpret as a positive sign of interdisciplinarity.

Quantifiable variables to measure the success of adaptation measures are not common (indicators are preferred over metrics, and units of measurement are often absent). While the greater attention to formative approaches (inputs and outputs) positively indicates the recognition of adaptation as a process rather than only an end goal, attention to summative approaches is also required, to allow adaptation actors to understand the performance, efficiency, effectiveness, equity and sustainability of adaptation interventions^12^. A longer-term view is essential to cope with the uncertainty related to the impacts of climate change and adaptation ambiguities^7,45,46^ and to plan for transformative adaptation and broader change^47^. However, the context-adjustment requirements and the mismatch between the timescale of an adaptation intervention and the time taken for the intervention results to become evident are likely to be a challenge. When designing I&Ms, this limits the focus to process and short-term outcomes^7,22,38,48^, which is also observable in our study. A general lack of measurement units highlights the need for accurate and reliable data interpretation, particularly for impact indicators and those related to governance and institutional aspects. In relation to the latter, while policy document analyses reveal an abundance of governance-related I&M^12^, their limited treatment in the scholarly literature reviewed, also found elsewhere^49^, may reflect a lack of engagement with, or understanding of, local government needs and on-the-ground adaptation implementation realities.

To guarantee a strong focus on climate adaptation, in our study, we have excluded publications looking at general resilience or disaster risk reduction; however, we observe that the development of adaptation I&M is significantly influenced by their frameworks and models. This demonstrates the need to bridge climate change, disaster, and resilience agendas^50^. Previous studies show how common local resilience assessment tools and frameworks display an abundance of I&M^51^, which evidences the need for cross-examination and cross-fertilisation of resilience and adaptation MERL fields. Holistic approaches to capture multiple risks and interactions of different hazards are also lacking, which calls for greater attention to cascading and compound effects of a combination of hazards on urban systems and populations, beyond climate change^39,52^.

The information regarding end-users and reasons for measuring is generally vague and ambiguous, questioning how usable the proposed urban adaptation I&M are in real-world contexts beyond academia. A lack of attention to reasons to measure adaptation beyond accountability and performance assessment highlights the need for further theoretical and empirical work to explore how adaptation measurement matters for equity, finance, politics, and broader societal, biodiversity, and climate-related challenges.

Based on this systematic review, we conclude that many existing I&M methods are outdated and fail to capture the complexity of today’s urban climate adaptation challenges. Our work provides essential groundwork for developing more robust guidance on the design, implementation, and application of adaptation I&M. We highlight three critical areas for future research: quantifiability, long-term assessments, and policy focus. Beyond its scientific and technical value, this systematic effort has the potential to drive significant progress across adaptation governance levels, as both private and public organisations strengthen and institutionalise their adaptation monitoring, evaluation, reporting, and learning processes.

## Methods

### Monitoring, Evaluation, Reporting and Learning (MERL)

Also referred to as M&E or MEL. With the implementation of adaptation interventions, there is a clear need to monitor, evaluate, report, and learn from actions to follow and assess progress, as well as identify good practices. The terms “monitoring”, “evaluation”, “reporting”, and “learning”, collectively referred to as “MERL”, make up different parts of this process. “Monitoring” refers to ongoing data collection in a systematic manner, typically through I&M, whereas “Evaluation” refers to assessments that usually occur at predefined intervals^13^. “Reporting” and “Learning” are often implied within the monitoring and evaluation process, with “reporting” referring to the processes in places for accountability and communication of results, and “learning” focusing more explicitly on measures and information used to assess “are we doing the right things” and identify areas in need of improvement^13,38^.

### Indicators and Metrics (I&M)

Indicators and metrics are key components common to most MERL systems. However, as noted previously, the terms “indicator” and “metric” are often used and expressed interchangeably as “I&M”, and the lack of clarity between these two terms^11^ is widely documented and discussed. Here, an “indicator” is taken as a quality or trait that suggests a trend or “indicates” the effectiveness, progress, or success of what is being measured. In practice, this may include changes in behaviour, the orientation of buildings, the existence of a separate walking lane, or changes in living standards or awareness. Whilst all these factors are measurable, they cannot be readily quantified or tracked. By contrast, the term “metric” refers to a specific variable that can unambiguously be measured (if quantifiable) or tracked (if qualitative). Examples of metrics may include mortality rate, per capita income, built-up area, or peak flow rate.

### Inputs, outputs, outcomes and impacts

Adaptation I&M are either process-based or result-based. Process-based ones track the enabling environment for adaptation interventions or specific outputs resulting from the intervention itself. In this study, based on existing approaches to adaptation I&M^10,12^, we categorise process-based I&M as either “input”, referring to the capacity or resources used for adaptation in the enabling environment, or “output”, the direct quantitative success of project activities or products. There are broader definitions of input indicators (see Pearce-Higgins et al.^34^) that include enabling conditions or existing adaptive capacities. These have also been considered. Target indicators, as defined by Hale et al.^10^ are less applicable to this scientific context. “Inputs” indicators typically measure financing, staff availability, or the number of workshops conducted, whereas “outputs” may include hectares of land restored, an increase in green area, the number of projects delivered, or implementation of a plan or piece of legislation. Result-based I&M track the wider effects or long-term impact of an intervention and are either outcomes that reflect the visible short- to medium-term effects on ecological, economic, or social systems, or “impact” that reflects the long-term impact over decades or centuries. Typically, “outcomes” measure changes such as a reduction in flooding or an increase in thermal comfort, whereas “impacts” refer to the longer-term changes such as living standards, levels of poverty, or health.

### Methods

Between February 2022 and June 2023, we performed a systematic review and analysis of publications and indicators and metrics (I&M) found in scientific literature. We analysed scientific publications from the LENS scholarly literature database www.lens.org, which is openly accessible and diverse in the typologies of scientific publications. An original search provided 838 records, from which we selected and analysed 137 based on our inclusion and exclusion criteria (Screening and Coding Stage 1). We then collected and analysed 901 I&M (including indices) (Screening and Coding Stage 2). Eleven analysts participated in Stage 1, and 12 analysts participated in Stage 2.

The first step involved setting the scope of the review work. This scope later guided the use of the keyword search in the literature database and the identification of the publication inclusion and exclusion criteria (see Tables S1 and S2). The whole review process is summarised in Fig. S1. We included publications related to the urban scale or having urban implications; publications related to adaptation to climate change, but not resilience, sustainability or DRR in general, without a specific focus on climate change adaptation, and we aimed for publications including at least one indicator or metric. We only gathered publications in the English language to enable cross-review of collected data by the international team of analysts.

### Keywords string used

Scholarly Works (838) = title:((adapt* OR resilien*) AND (indicator* OR metric* OR index OR indic* OR eval* OR assess* OR measur* OR track* OR monitor*)) AND (title:((climat*) AND (urban* OR municipal* OR city OR cities OR metropolitan*)) OR abstract:((climat*) AND (urban* OR municipal* OR city OR cities OR metropolitan*)) OR keyword:((climat*) AND (urban* OR municipal* OR city OR cities OR metropolitan*)) OR field_of_study:((climat*) AND (urban* OR municipal* OR city OR cities OR metropolitan*))) AND (title:(NOT seismic* NOT earthquake* NOT tsunami*) OR abstract:(NOT seismic* NOT earthquake* NOT tsunami*) OR keyword:(NOT seismic* NOT earthquake* NOT tsunami*) OR field_of_study:(NOT seismic* NOT earthquake* NOT tsunami*)).

LENS Static Collection used for this review showed 838 records as of 15 February 2022 https://www.lens.org/lens/search/scholar/list?collectionId=199042. The LENS Dynamic Collection connected to the static collection and the same keywords string, shows 1164 records as of 29 December 2023, reflecting a 30% increase in publications in the field1164. https://link.lens.org/tGULKZKDMAj.

The next step consisted of the development of a coding protocol (or documenting protocol) for both publications and indicators. Tables 1 and 2 provide a summary of the main areas documented in both instances. After data collection, there was an intense process of data curation and analysis that led to a re-categorisation of data for analysis purposes and interpretability.

**Table 1:** Summary of main areas that have been documented for each publication

	Metadata category	Description
a	Disciplinary background of Lead Author (Free text)	Disciplinary background of the lead author using keywords selected by the author in Official institutional websites, Research Gate or Google Scholar.
b	Type of study (Checkbox)	Empirical, Theoretical or Review.
c	Research Purpose (Checkbox)	Descriptive/Exploratory, Explanatory, Evaluative
d	Research approach (Checkbox)	Type of methods used for analysis: Qualitative, Quantitative or Mixed methods.
e	Location of case study (Checkbox)	Region of the world where the study area for the research is located, e.g. North America, Latin America, Europe, Africa, Asia, Oceania.
f	Specific location (Free text)	Name of the city or cities and country where the study area for the research is located.
g	Scale of study (Checkbox)	Geographical extent of study in the region identified in (f) above: Supralocal, Metropolitan area, Urban agglomeration, Peri-urban, City, District, Neighbourhood, Household or community level.
h	Climate Risks (Checkbox)	Climate risks addressed: Adaptation to rain/river flooding, Adaptation to storm / coastal flooding, Adaptation to heatwaves, Adaptation to water security, Adaptation to food security.
i	Adaptation measure/sector (Checkbox)	Category of adaptation measure(s) addressed in the publication using IPCC categorisation. Land-use planning, Livelihoods and social protection, Emergency management and security, Health, Education & Comms, Cultural heritage and institutions, Temperature regulation, Air quality regulation, Stormwater and sanitation, Coastal flood protection, Riverine flood impact reduction, Water provisioning and management, Food production and security, Built form, Housing and building design, ICT (information, communication and technology), Energy infrastructures, Transport, Water and sanitation, Flood management, Coastal management.
j	Purpose of the evaluation (Checkbox)	Formative or Summative. Studies focusing on formative assessment involve ex-ante evaluation and continuous monitoring of the conditions from the early stages of the planning process. Studies focusing on summative assessment involve an ex-post measure of the effectiveness of interventions.
k	Theoretical Framework (Free text)	Theoretical framework or evaluation theory used in the publication to develop and define indicators and metrics, or their frameworks.
l	Intended user or audience (Checkbox)	Target audience of the research explicitly mentioned or inferred from the text. Scientific community, Local authorities, Urban planners, Local actors in general, Financial actors, Private companies, Citizens, Regional or national government bodies, International city networks (reporting systems), Non-governmental organisation (NGO) or advocacy groups.

**Table 2:** Data collected for each indicator or metric identified in the literature

	Datacollected	Description
a	Name (Free text)	Name of the indicator/metric as indicated in the document.
b	Composite nature (Checkbox)	Whether the variable is an index, i.e. a composite indicator. Yes or No.
c	Level of detail (Checkbox)	Tangibility in two levels: Indicator/ Metric.
d	Type (Checkbox)	The type of I&M identified in (c). Input, Output, Outcome or Impact.
e	Adaptation Measure (if applicable) (Checkbox)	Whether there is a specific adaptation action/measures/policy connected to the indicator.
f	Dimension (Checkbox)	Domains evaluated or monitored by the indicator. Social/ human/ society, Economic/finance, Environmental/natural, Built infrastructure, Governance/institutional/political.
g	Spatial scale of the indicator (Checkbox)	The scale to which data for this indicator is collected. Supralocal, City, District, Neighbourhood, Household, community or individual, Street or building.
h	Data Source (Checkbox)	Source of the data. Survey, Interviews and focus groups, Historical and statistical data, Spatial data and statistical observations, Expert and literature data.
i	Unit of measurement (Free text)	Unit of measurement assigned to the indicator or metric.
j	Frequency of measurement (Free text)	Frequency of measurements to be carried out to monitor the indicator or metric.
k	Applicability of indicator (Checkbox)	The applicability or use of the adaptation data that will be collected through the I&M. Assess adaptation needs and their dynamics/evolution (including changing vulnerabilities or risks, Assess efficiency of adaptation efforts and processes, Provide accountability of adaptation actions (direct outputs), Assess results (outcomes and impacts) of adaptation actions, Understand equity of adaptation progress and justice of adaptation, Improve future adaptation activities or interventions, Compare with other similar adaptation activities or interventions, Attract funding and distribute resources, Gather political momentum, Increase understanding of adaptation and its relationship with urban development, sustainability and other societal challenges.

## Supplementary information

NPJURBANSUSTAIN-01070-T Supplementary Information

## Data Availability

Data generated or analysed during this study are included in this published article (and its Supplementary Information) and online repositories. The information available through online repositories includes the dataset of publications and indicators and connected metadata, which can be found online at DOI [10.5281/zenodo.10663610] (10.5281/zenodo.10663610).

## References

[1] Peters, G. P. et al. Key indicators to track current progress and future ambition of the Paris Agreement. *Nat. Clim. Change* **7**, 118–122 (2017).

[2] New, M. et al. Decision-Making Options for Managing Risk. In *Proc. Climate Change 2022: Impacts, Adaptation and Vulnerability. Contribution of Working Group II to the Sixth Assessment Report of the Intergovernmental Panel on Climate Change* (eds. Pörtner, H.-O. et al.) 2539–2654 10.1017/9781009325844.026 (Cambridge University Press, 2022).

[3] Berrang-Ford, L. et al. A systematic global stocktake of evidence on human adaptation to climate change. *Nat. Clim. Change* **11**, 989–1000 (2021).

[4] Eriksen, S. et al. Adaptation interventions and their effect on vulnerability in developing countries: help, hindrance or irrelevance?. *World Dev.* **141**, 105383 (2021).

[5] Sparkes, E. & Werners, S. E. Monitoring, evaluation and learning requirements for climate-resilient development pathways. *Curr. Opin. Environ. Sustain.* **64**, 101329 (2023).

[6] Fisher, S. Much ado about nothing? Why adaptation measurement matters. *Clim. Dev.* **16**, 161–167 (2024).

[7] Goodwin, S., Olazabal, M., Castro, A. J. & Pascual, U. Measuring the contribution of nature-based solutions beyond climate adaptation in cities. *Glob. Environ. Change* **89**, 102939 (2024).

[8] Amorim-Maia, A. T. & Olazabal, M. Beyond adjustment: a new paradigm for climate change adaptation in a complex world. *Glob. Environ. Change* **93**, 103027 (2025).

[9] Nalau, J. & Verrall, B. Mapping the evolution and current trends in climate change adaptation science. *Clim. Risk Manag*. 100290 10.1016/j.crm.2021.100290 (2021).

[10] Hale, T. N. et al. Sub- and non-state climate action: a framework to assess progress, implementation and impact. *Clim. Policy* **21**, 406–420 (2021).

[11] Arnott, J. C., Moser, S. C. & Goodrich, K. A. Evaluation that counts: a review of climate change adaptation indicators & metrics using lessons from effective evaluation and science-practice interaction. *Environ. Sci. Policy* **66**, 383–392 (2016).

[12] Goonesekera, S. M. & Olazabal, M. Climate adaptation indicators and metrics: State of local policy practice. *Ecol. Indic.* **145**, 109657 (2022).

[13] Klostermann, J. et al. Towards a framework to assess, compare and develop monitoring and evaluation of climate change adaptation in Europe. *Mitig. Adapt. Strateg. Glob. Change* **23**, 187–209 (2018). 10.1007/s11027-015-9678-4 PMC6054010 30093829

[14] Leiter, T. Do governments track the implementation of national climate change adaptation plans? An evidence-based global stocktake of monitoring and evaluation systems. *Environ. Sci. Policy* **125**, 179–188 (2021).

[15] Beauchamp, E. & Józefiak, I. *Next Steps for Defining a Monitoring, Evaluation, and Learning System for the Global Goal on Adaptation by COP 28*. https://www.iisd.org/publications/report/global-goal-on-adaptation-monitoring-evaluation-learning-framework-cop-28 (2023).

[16] Canales, N., Klein, R. J. T., Bakhtaoui, I. & Macura, B. Assessing adaptation progress for the global stocktake. *Nat. Clim. Change* 1–2 10.1038/s41558-023-01656-x (2023).

[17] Adaptation Committee. *Approaches to Reviewing the Overall Progress Made in Achieving the Global Goal on Adaptation*. https://unfccc.int/process-and-meetings/bodies/constituted-bodies/adaptation-committee-ac/publications-bulletin-adaptation-committee (2021).

[18] Leiter, T. Too little, too slow? Climate adaptation at the United Nations climate change negotiations since the adoption of the Paris Agreement. *Carbon Clim. Law Rev.* **16**, 243–258 (2023).

[19] Dodman, D. et al. Cities, settlements and key infrastructure supplementary material. In *Proc. Climate Change 2022: Impacts, Adaptation, and Vulnerability. Contribution of Working Group II to the Sixth Assessment Report of the Intergovernmental Panel on Climate Change* (eds. Pörtner, H.-O. et al.) (IPCC, 2023).

[20] Berrang-Ford, L. et al. Tracking global climate change adaptation among governments. *Nat. Clim. Change* **9**, 440 (2019).

[21] Olazabal, M., Galarraga, I., Ford, J., Sainz de Murieta, E. & Lesnikowski, A. Are local climate adaptation policies credible? A conceptual and operational assessment framework. *Int. J. Urban Sustain. Dev.* **11**, 277–296 (2019).

[22] IPCC. *Climate Change 2022*: *Impacts, Adaptation, and Vulnerability*. *Contribution of Working Group II to the Sixth Assessment Report of the Intergovernmental Panel on Climate Change* (Cambridge University Press, 2023).

[23] Reckien, D. et al. Quality of urban climate adaptation plans over time. *npj Urban Sustain* **3**, 1–14 (2023).

[24] Biesbroek, R. & Lesnikowski, A. Unpacking symbolic policy-making for the first Global Stocktake under the Paris Agreement. *npj Clim. Action* **2**, 1–3 (2023).

[25] Olazabal, M., Ruiz de Gopegui, M., Tompkins, E. L., Venner, K. & Smith, R. A cross-scale worldwide analysis of coastal adaptation planning. *Environ. Res. Lett.* **14**, 124056 (2019).

[26] Solecki, W. & Rosenzweig, C. Indicators and monitoring systems for urban climate resiliency. *Clim. Change*10.1007/s10584-020-02947-4 (2020).

[27] Tyler, S. et al. Indicators of urban climate resilience: a contextual approach. *Environ. Sci. Policy* **66**, 420–426 (2016).

[28] Kabisch, N. et al. Nature-based solutions to climate change mitigation and adaptation in urban areas: perspectives on indicators, knowledge gaps, barriers, and opportunities for action. *Ecol. Soc.* **21**, 39 (2016).

[29] Sanchez Martinez, G., von der Pahlen, M. T., Kendrovski, V., Linares, C. & Diaz, J. Indicators to monitor policy progress in health adaptation to climate change: do they really do the job? *Eur. J. Public Health* **30**, Supplement_5 (2020).

[30] Salehi, S. et al. Climate change adaptation: a systematic review on domains and indicators. *Nat. Hazards* **96**, 521–550 (2019).

[31] Feldmeyer, D. et al. Indicators for monitoring urban climate change resilience and adaptation. *Sustainability* **11**, 2931 (2019).

[32] Granceri Bradaschia, M., Longato, D., Maragno, D. & Musco, F. Climate change adaptation mainstreaming through strategic environmental assessments. An in-depth analysis of environmental indicators from spatial plans in Friuli Venezia Giulia Region (Italy). *Environ. Impact Assess. Rev.* **109**, 107650 (2024).

[33] Chmutina, K., Lizarralde, G., von Meding, J. & Bosher, L. Standardised indicators for “resilient cities”: the folly of devising a technical solution to a political problem. *Int. J. Disaster Resil. Built Environ.* **14**, 514–535 (2023).

[34] Pearce-Higgins, J. W. et al. A framework for climate change adaptation indicators for the natural environment. *Ecol. Indic.* **136**, 108690 (2022).

[35] Kuhlicke, C. et al. Spinning in circles? A systematic review on the role of theory in social vulnerability, resilience and adaptation research. *Glob. Environ. Change* **80**, 102672 (2023).

[36] Lindner, R., Hernantes, J. & Jaca, C. Foster the application of ISO standards on climate change adaptation in cities. *J. Stand*. **3**, 1 (2024).

[37] J. B. A. Consulting. *Review of Climate Resilience Mainstreaming into Regulatory and Voluntary Standards, National Guidance, and Other Sectoral/Industry Codes of Practice*. https://www.ukclimateresilience.org/projects/review-of-standards-guidance-and-codes-of-practice-for-enhancing-climate-resilience/ (2020).

[38] Pringle, P. & Leiter, T. Pitfalls and potential of measuring climate change adaptation through adaptation metrics. In *Proc. Adaptation metrics: Perspectives on measuring, aggregating and comparing adaptation results* (eds. Christiansen, L., Martinez, G. & Naswa, P.) 29 (UNEP DTU, 2018).

[39] Laurien, F., Martin, J. G. C. & Mehryar, S. Climate and disaster resilience measurement: persistent gaps in multiple hazards, methods, and practicability. *Clim. Risk Manag.* **37**, 100443 (2022).

[40] Vincent, K. & Cundill, G. The evolution of empirical adaptation research in the global South from 2010 to 2020. *Clim. Dev.* **14**, 25–38 (2022).

[41] Zall Kusek, J. & Rist, R. C. *Ten Steps to a Results-Based Monitoring and Evaluation System: A Handbook for Development Practitioners* (World Bank Group, 2004).

[42] OECD. *Measuring Progress in Adapting to a Changing Climate: Insights from**OECD Countries*. 10.1787/8cfe45af-en (OECD, 2024).

[43] Olazabal, M. & Ruiz De Gopegui, M. Adaptation planning in large cities is unlikely to be effective. *Landsc. Urban Plan.* **206**, 103974 (2021).

[44] Ruiz de Gopegui Aramburu, M., Olazabal, M. & Broto, V. C. Examining climate justice in urban public space adaptation: a thematic synthesis of the literature. *J. City Clim. Policy Econ.* **2**, 271–315 (2024).

[45] Brugnach, M. & Hoek, R. van den. Embracing ambiguity in climate change adaptation for more effective responses to new uncertain shorescapes conditions. *Mar. Policy* **152**, 105626 (2023).

[46] Bartelet, H. A., Barnes, M. L., Bakti, L. A. A. & Cumming, G. S. Operationalizing and measuring climate change adaptation success. *Ecol. Soc*. **30**, 1 (2025).

[47] Revi, A. et al. Transformative adaptation in cities. *One Earth* **3**, 384–387 (2020).

[48] Brooks, N. & Fisher, S. *Tracking Adaptation and Measuring Development: A Step-by-Step Guide*. http://pubs.iied.org/10100IIED.html (2014).

[49] Dupuits, E., Garcés, A., Llambí, L. D. & Bustamante, M. Strategies for monitoring and evaluation of climate change adaptation: localizing global approaches into Andean realities. *npj Clim. Action* **3**, 19 (2024).

[50] Sainz de Murieta, E., Galarraga, I. & Olazabal, M. How well do climate adaptation policies align with risk-based approaches? An assessment framework for cities. *Cities* 103018 10.1016/j.cities.2020.103018 (2020).

[51] Sharifi, A. A critical review of selected tools for assessing community resilience. *Ecol. Indic.* **69**, 629–647 (2016).

[52] Westman, L. et al. Compound urban crises. *Ambio* **51**, 1402–1415 (2022). 35157255 10.1007/s13280-021-01697-6 PMC8853022

[53] Turner, S., Moloney, S., Glover, A. & Fünfgeld, H. *A Review of the Monitoring and Evaluation Literature for Climate Change Adaptation* (RMIT University Centre for Urban Research, 2014).

