# Supplementary material for: Conventional approaches to indicators and metrics undermine urban climate adaptation
Source: NPJ Urban Sustain. 2025 Dec 14;6(1):8. doi: 10.1038/s42949-025-00310-z (PMC12795751; doi:10.1038/s42949-025-00310-z)
Supplement: Supplementary file 1 — NPJURBANSUSTAIN-01070-T Supplementary Information [file 42949_2025_310_MOESM1_ESM.pdf]

Supplementary Information

Table S1. Summary of inclusion and exclusion criteria for publication records.

**INCLUSION CRITERIA (must meet all)**	**EXCLUSION CRITERIA (meeting one criterion equals exclusion from the final list)**
TYPES OF PUBLICATIONS: scientific articles, thesis, books, book chapters and conference proceedings that present research work on urban adaptation assessment.	LANGUAGE: Is the publication in English? Publications in any language other than English will be excluded.
PRESENCE OF INDICATORS: Publications need to suggest/propose/discuss at least one specific indicator/metric/measurable objective.	DUPLICATION: Is the publication duplicated? When there is a journal article and a conference proceeding or book chapter with the same contents, it is considered a duplication.
URBAN SCALE: The evaluation, which is addressed in the publication, needs to look at adaptation in general or specific adaptation interventions at the local urban scale. This includes local, metropolitan and the building scale.	ACCESSIBILITY: Is the full text or required text for screening not accessible?
ADAPTATION: The specific framing of this evaluation in the context of climate change adaptation needs to be clear (including adaptation to any direct or indirect climate-induced hazard or combinations).	RESILIENCE: Is the Focus on resilience in general? If the focus of the paper is not climate change but resilience in general, despite climate change being mentioned as a potential shock, it must be excluded.
	DISASTERS: Is the Focus on disasters in general? If the focus of the paper is on disasters in general, despite climate change being mentioned as a potential disaster, it must be excluded.
	URBAN IMPLICATIONS: Are implications in/of the urban context disregarded? Test question: Could this intervention be implemented in a rural area? If yes, then it should not be included.

Table S2. Summary of exclusion criteria used to select indicators and metrics (I&M).

**EXCLUSION CRITERIA (meeting one criterion equals exclusion from the final list)**
1. Publication is excluded from the analysis in Stage 2
2. The indicator, metric or index is not clearly listed or has a clear identifier.
3. The indicator or metric focuses on evaluating other aspects of climate action (i.e. mitigation) or sustainability/resilience without a claimed connection to adaptation.
4. The indicator or metric is part of a broader index (in this case, only consider the index)

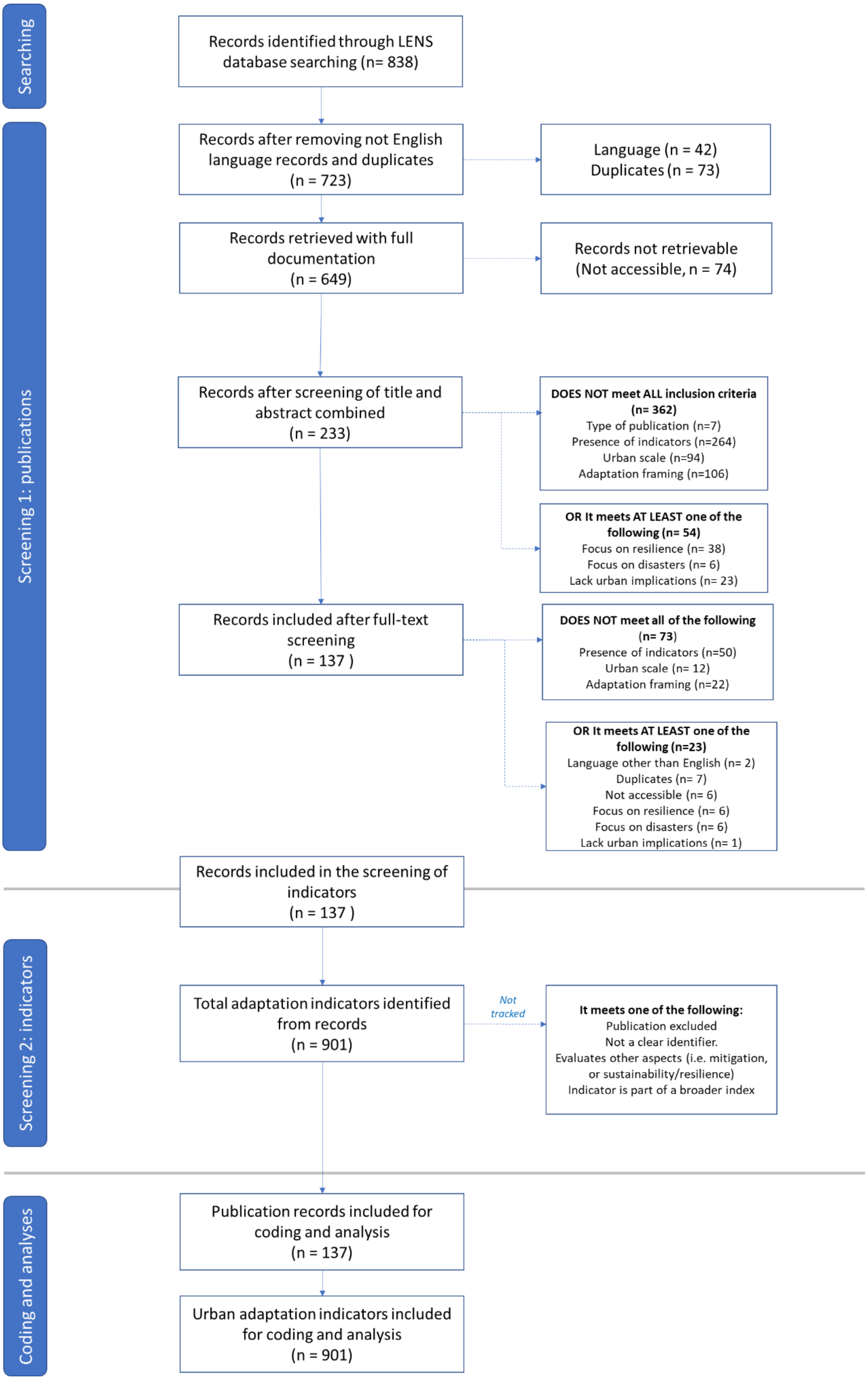

Figure S1. The Roses diagram illustrates the process of screening and coding of publications and indicators in our systematic review. Adapted from Haddaway et al.[38]. The total number of excluded indicators is not accounted for.
